# Central Venous Catheter Tracking Into a Persistent Left Superior Vena Cava in a Patient With Complete Heart Block

**DOI:** 10.7759/cureus.105042

**Published:** 2026-03-11

**Authors:** Ahmed R Abdelwahed, Andrew Issa, Bradley Chuchian, Alan Orellana, Vinay Shet

**Affiliations:** 1 Internal Medicine, Mountain View Regional Medical Center, Las Cruces, USA; 2 Critical Care, Mountain View Regional Medical Center, Las Cruces, USA

**Keywords:** central venous line, complete heart block (chb), coronary sinus (cs), leadless pacemaker implantation, persistent left superior vena cava (plsvc)

## Abstract

Persistent left superior vena cava (PLSVC) is the most common thoracic venous anomaly, present in approximately 0.3-0.5% of the general population. Although typically asymptomatic, PLSVC carries significant procedural implications during central venous catheter (CVC) placement, pacemaker implantation, and hemodynamic monitoring.

A 75-year-old man presented with symptomatic complete heart block and was transferred for pacemaker evaluation. A right internal jugular (IJ) central venous catheter was inserted for vasopressor administration. Post-procedure chest X-ray revealed the catheter coursing along the left mediastinum instead of crossing the midline. Bedside ultrasound confirmed venous placement, and arterial versus venous blood gas comparison supported correct venous cannulation. Further cardiology evaluation, echocardiography, and cardiac catheterization identified a PLSVC, explaining the aberrant catheter trajectory.

Awareness of PLSVC is essential to avoid misinterpreting catheter malposition as arterial puncture or mediastinal injury. Recognizing characteristic radiographic findings can prevent unnecessary line removal and support safe procedural decision-making.

## Introduction

Persistent left superior vena cava (PLSVC) happens when the left anterior cardinal vein does not regress during embryonic development. It is the most common thoracic venous anomaly, found in about 0.3-0.5% of people overall and up to 10% of those with congenital heart disease [1]. While it usually does not cause symptoms, it becomes important during procedures like central venous access, pacemaker or implantable cardioverter-defibrillator (ICD) placement, thoracic surgery, and right-sided heart catheterization. Most often, the PLSVC drains into the coronary sinus, which can become enlarged, and to a lesser extent, into the left atrium, increasing the risk of arrhythmias, creating a right-to-left shunt, or complications during lead placement [2].

## Case presentation

A 75-year-old man with type 2 diabetes, chronic kidney disease, high blood pressure, high cholesterol, and sleep apnea was transferred for evaluation of symptomatic complete heart block. He had several days of worsening dizziness and lightheadedness, and his heart rate was in the 30s at the referring hospital.

On arrival, he was awake and alert but had significant hemodynamic instability, with blood pressures ranging from 178/76 mmHg to 76/35 mmHg. Lab tests showed acute kidney injury (creatinine 2.7 to 3.5 mg/dL), worsening lactic acidosis (2.7 to 10.6 mmol/L), a rising white blood cell count (11.33 to 22.15 K/µL), and an increasing high-sensitivity troponin (346.13 to 15,424 ng/L), as can be seen in Table 1, consistent with a supply-demand mismatch.

**Table 1:** Laboratory findings on arrival and the next day and the reference range

Laboratory Test	Patient Values on 12/02/2025	Patient Values on 12/03/2025	Reference Range
Creatinine	2.7 mg/dL	3.5 mg/dL	0.5–1.5 mg/dL
Lactic Acid	2.7 mmol/L	10.6 mmol/L	0.5–1.9 mmol/L
White Blood Cell Count (WBC)	11.33 K/µL	22.15 K/µL	4.23–9.07 K/µL
High-Sensitivity Troponin	346.13 ng/L	15,424 ng/L	0.00 - 53.48 ng/L
Glucose	78 mg/dL	28 mg/dL	74–106 mg/dL

Since he needed vasopressor support, a right internal jugular central venous catheter was placed with ultrasound guidance. Venous access was confirmed by watching the needle enter the internal jugular (IJ) vein and by aspirating nonpulsatile, dark blood.

**Figure 1:**
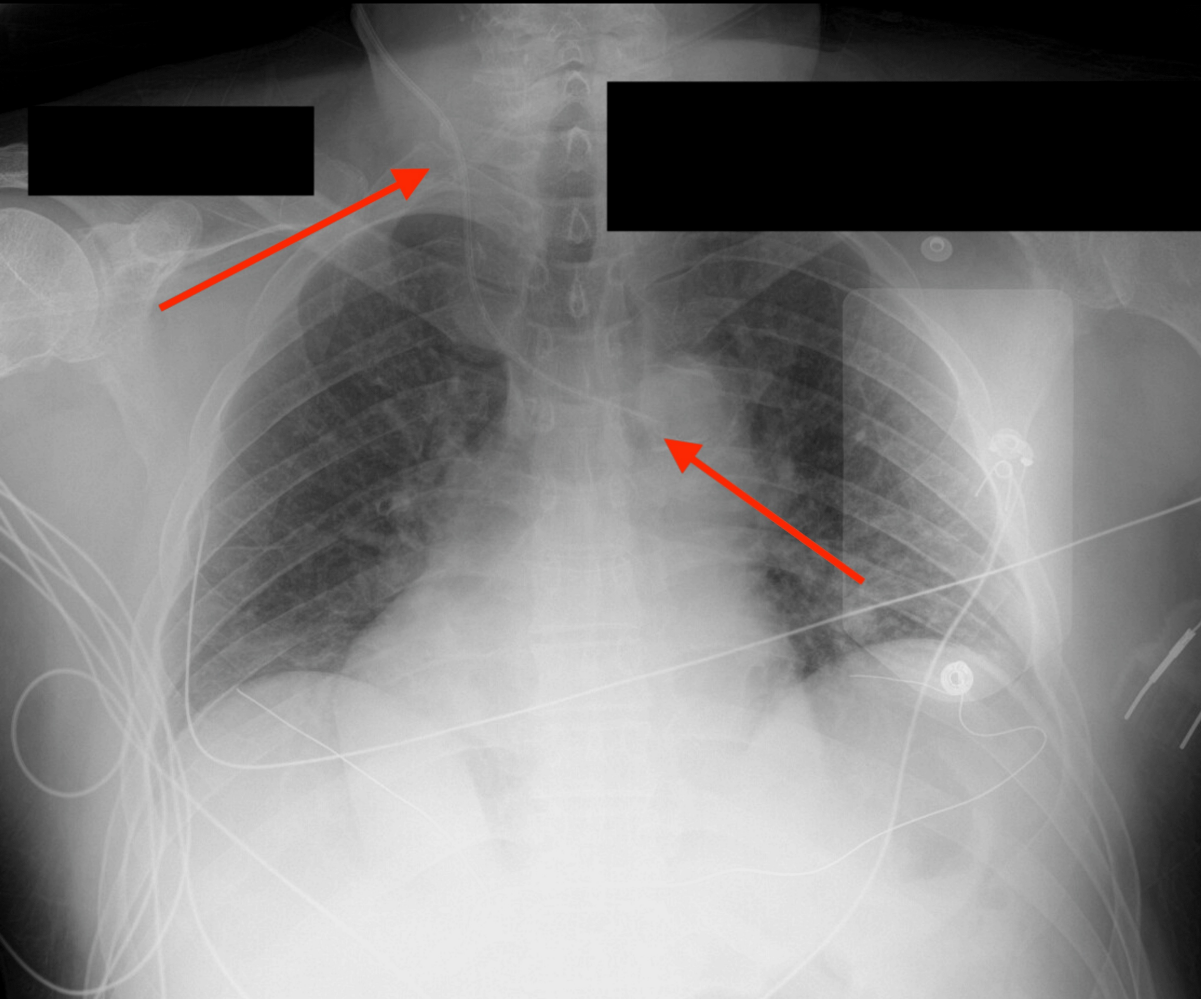
Initial central venous catheter insertion The initial central venous catheter was inserted from the right side of the neck and then crossed to the left side, as shown by the arrows.

A chest X-ray after the procedure showed something unexpected: the catheter crossed to the left side of the mediastinum instead of running vertically toward the right atrium, as can be seen in Figure 1. Possible explanations included arterial cannulation, mediastinal malposition, dextrocardia, or an unusual vein, such as a PLSVC.

**Figure 2:**
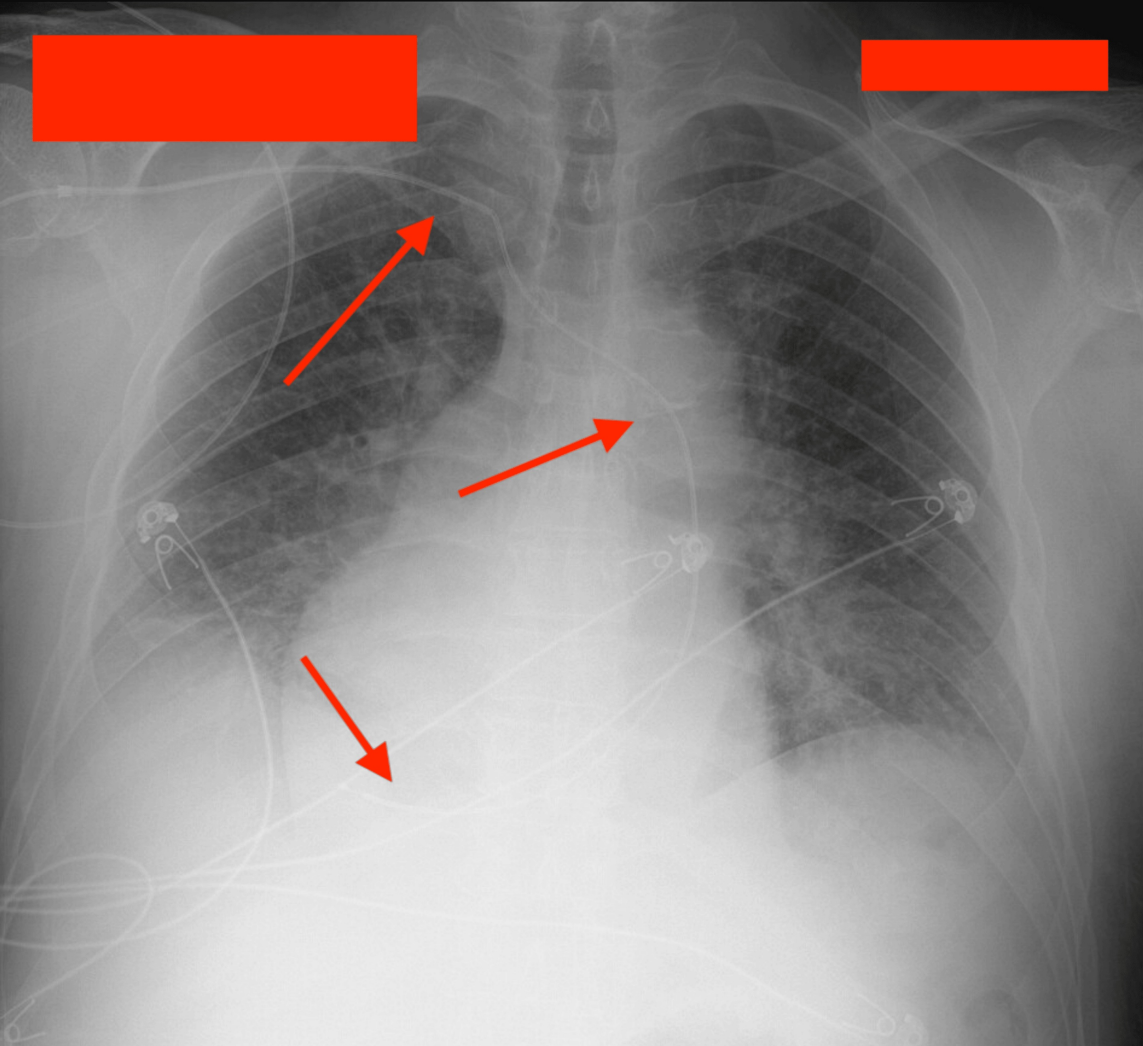
Central venous catheter with the right subclavian approach A central venous catheter and a guide wire appear to be advanced by the cardiologist through the right subclavian vein, crossing to the left side before returning to the right side, as shown with arrows.

**Figure 3:**
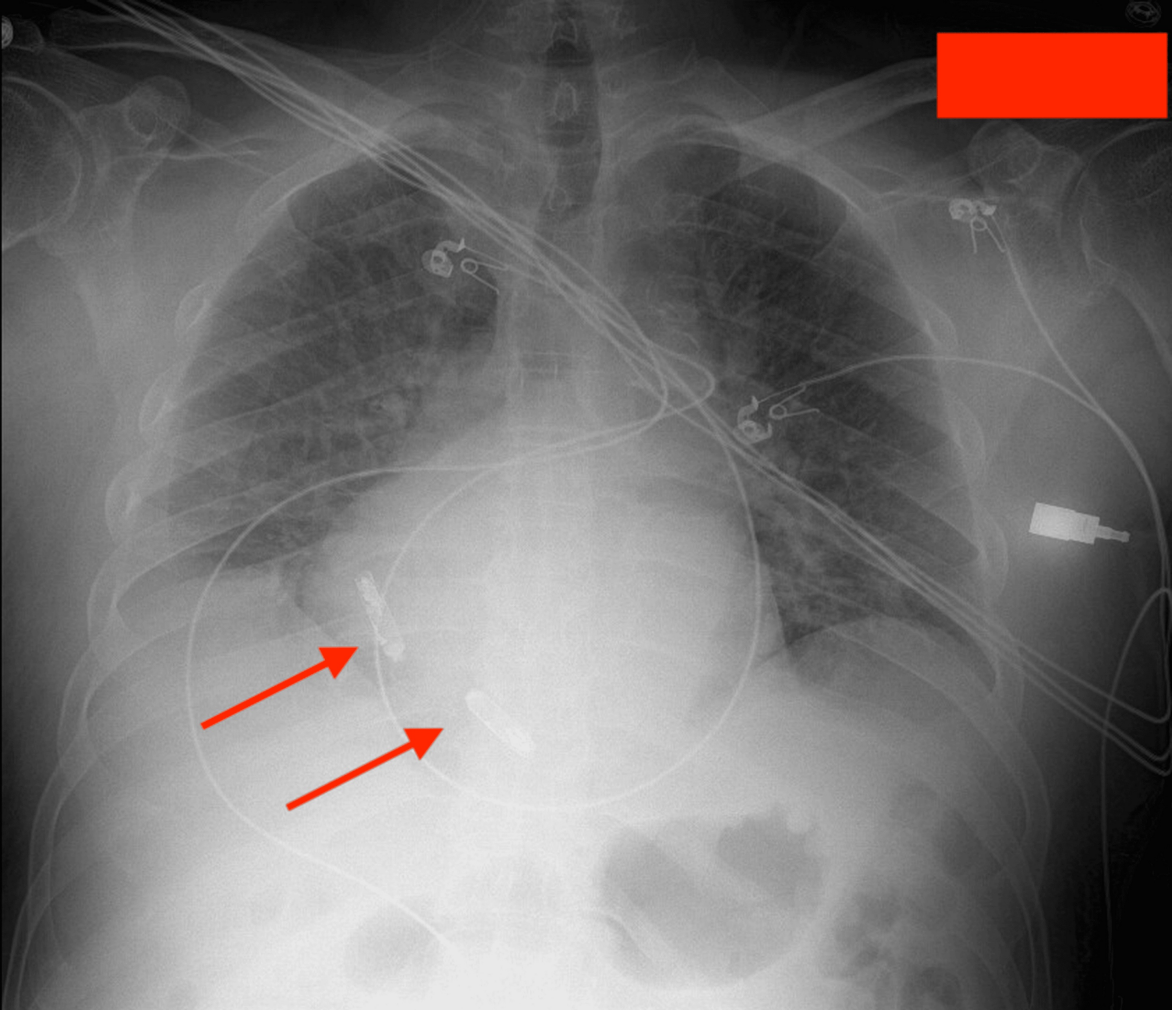
Leadless pacemaker A leadless pacemaker was placed by the cardiologist, as shown in the figure with arrows.

The cardiology team did further evaluation. Transthoracic echocardiography showed a very dilated coronary sinus, raising suspicion for PLSVC. Cardiac catheterization confirmed a left-sided superior vena cava (SVC) with normal coronary anatomy and preserved left ventricular hemodynamics, as can be seen in Video 1 and Figure 2. After the left heart catheterization, the cardiologist's report read as normal left ventricular hemodynamics with abnormal left-sided vena cava, as can be seen in Video 1. Temporary transvenous pacing was done successfully through a femoral access. Taking into consideration the unusual venous anatomy, electrophysiology chose to place a leadless permanent pacemaker, as can be seen in Figure 3.

**Video 1:** Left heart catheter Video showing the left heart catheterization performed by the cardiologist.

After pacing, the patient’s blood pressure stabilized. Troponin levels decreased from 15,424 to 6,157 ng/L, and kidney function improved (creatinine fell to 1.8 mg/dL), as shown in Table 2. He had one episode of low blood sugar (glucose 28 mg/dL), as can be seen in Table 2, which was quickly treated with intravenous dextrose. There were no complications from the central venous catheter, and the rest of his hospital stay went smoothly.

**Table 2:** Lab values after a cardiac pacemaker was placed Lactic acid was 1.4 on 12/04/2025; therefore, it wasn't followed up on.

Laboratory Test	Patient Values on 12/06/2025	Reference Range
Creatinine	1.8 mg/dL	0.5–1.5 mg/dL
White Blood Cell Count (WBC)	8.03 K/µL	4.23–9.07 K/µL
High-Sensitivity Troponin	6157.73 ng/L	0.00 - 53.48 ng/L
Glucose	91 mg/dL	74–106 mg/dL

## Discussion

PLSVC is found in about 0.3-0.5% of the general population and up to 10% of people with congenital heart disease [1]. In most cases, it drains into the coronary sinus [3]. During right IJ catheterization, unusual venous pathways can cause the catheter to travel along the left mediastinum, which may appear as arterial misplacement or mediastinal injury. Recognizing this pattern is important to avoid unnecessary catheter removal or extra procedures. PLSVC can also make pacemaker and ICD placements more difficult because standard leads must pass through the coronary sinus, increasing the risk of perforation or instability [4]. That is why a leadless pacemaker was chosen for this patient. The patient’s son was reportedly “born with three heart chambers,” denoting a major congenital heart problem, likely single-ventricle physiology. PLSVC often coexists with congenital heart defects, including atrioventricular septal defects, univentricular hearts, heterotaxy syndromes, and systemic venous return anomalies [5]. While PLSVC itself is usually sporadic, it is well-documented that congenital cardiac anomalies can cluster in families [6]. Both mentioned conditions result from disruptions in overlapping embryologic pathways, especially those involved in venous development and cardiac septation [7]. So, having severe congenital heart disease in a first-degree relative makes a shared developmental susceptibility more likely, even if there is no direct hereditary pattern.

## Conclusions

This case shows why it is important to think about PLSVC when a right IJ catheter unexpectedly travels along the left side of the mediastinum. Recognizing this early might prevent unnecessary procedures, support safe planning, and inform decisions about pacemaker placement. A family history of congenital heart defects may be linked to PLSVC and should raise suspicion when evaluating unusual venous pathways.
